# Molecular Simulation Study on the Microscopic Structure and Mechanical Property of Defect-Containing sI Methane Hydrate

**DOI:** 10.3390/ijms20092305

**Published:** 2019-05-09

**Authors:** Shouyin Cai, Qizhong Tang, Sen Tian, Yiyu Lu, Xuechao Gao

**Affiliations:** 1 Key Laboratory of Low-grade Energy Utilization Technologies & Systems, Ministry of Education, College of Energy and Power Engineering, Chongqing University, Chongqing 400044, China; shouyincai@cqu.edu.cn; 2 State Key Laboratory of Coal Mine Disaster Dynamics and Control, School of Resources and Environmental Science, Chongqing University, Chongqing 400044, China; 20153113022@cqu.edu.cn (Q.T.); luyiyu@cqu.edu.cn (Y.L.); 3 State Key Laboratory of Materials-Oriented Chemical Engineering, College of Chemical Engineering, Nanjing Tech University, Nanjing 210009, China

**Keywords:** methane hydrate, defect, mechanical property, molecular dynamics (MD), order parameter

## Abstract

The study of changes in the related mechanical property and microscopic structure of methane hydrate during the decomposition process are of vital significance to its exploitation and comprehensive utilization. This paper had employed the molecular dynamics (MD) method to investigate the influence of defects on the microscopic structure and mechanical property of the sI methane hydrate system, and to discover the mechanical property for the defect-containing hydrate system to maintain its brittle materials. Moreover, the stress-strain curve of each system was analyzed, and it was discovered that the presence of certain defects in the methane hydrate could promote its mechanical property; however, the system mechanical property would be reduced when the defects had reached a certain degree (particle deletion rate of 9.02% in this study). Besides, the microscopic structures of the sI methane hydrate before and after failure were analyzed using the F3 order parameter value method, and it was found that the F3 order parameters near the crack would be subject to great fluctuations at the time of failure of the hydrate structure. The phenomenon and conclusions drawn in this study provide a basis for the study of the microscopic structure and mechanical characteristics of methane hydrate.

## 1. Introduction

Methane hydrate [1,2] (hereinafter referred as “hydrate”), which is generally distributed in the abyssal deposit or the permafrost of land area, is the glacial crystalline material formed by methane molecule with water molecule under high-pressure and low-temperature conditions. As one of the future new energies, hydrate is associated with the superiorities of little pollution, great reserves, wide distribution, and high energy density. It is estimated by the related department that the organic carbon reserve in the global hydrate is 1 × 10^16^ kg [3], which is 2-times the sum of all coal, oil, and natural gas resources discovered on the earth so far. At present, over 130 kinds of compounds have been identified to form the hydrates with water molecule; typically, the existing hydrates can be classified according to the crystal configuration as sI [4], sII [5], and sH type [6]. The most commonly seen methane hydrate generally exists in sI type in nature, which was chosen to be the object of this study.

Currently, the hydrate technique has been extensively applied in fields such as gas storage and transport, mixed gas separation and abyssal storage of carbon dioxide [7,8,9,10]. Generally, the storage conditions of hydrate will be changed during the exploitation process, while pressure reduction and temperature increase will accelerate the decomposition of hydrate [11,12,13]. On the one hand, the excessive release of methane gas after decomposition will accelerate the greenhouse effect; on the other hand, decomposition will change the mechanical property of the hydrate settled layer, and the hydrate at solid state will be decomposed into the liquid water and methane gas, which will reduce the shear strength of the settled layer and weaken the stratum stability, thus inducing severe geological damage [14]. Consequently, it is of vital significance to investigate the related changes in the mechanical property and microscopic structure at the time of hydrate decomposition for its exploitation and comprehensive utilization. So far, several studies on mechanical properties of the methane hydrate have been done. Winters et al. and Waite et al. [15,16] had investigated the mechanical properties of water-bearing sediments in permafrost and marine areas, and discovered that the various pore fillers would affect the strength and pore pressure of the hydrate. Ruppel et al. [17,18,19] had adopted the tri-axial test to compare and analyze various mechanical properties of the experimentally synthesized and natural hydrates, including the bulk modulus, stress-strain curve, Poisson’s ratio, and shear strength. Kuniyuki et al. [20] discovered that the elastic modulus of the hydrate was sharply decreased with the decomposition of hydrate, which had produced great transient changes. Masayuki et al. [21] had analyzed the relationships of methane hydrate failure degree with temperature, pressure, and hydrate saturation through the tri-axial compression experiment; their research suggested that the shear strength and elastic modulus of the test specimen tended to be increased with the increase in hydrate saturation. Typically, the mechanical property of hydrate plays a decisive role in the mechanical property of the sediment [22].

The experiment to test the hydrate mechanical property is demanding on the used equipment, moreover, the synthesized hydrate in the experiment usually contains lattice defect or cavity and other impurities, which have resulted in great difficulties in investigating the mechanical property of hydrate through experiment. The molecular dynamic (MD) simulation method [23,24,25,26,27,28,29,30] can intuitively reappear the molecular trajectory, which has thus been extensively applied in studying the microscopic substance mechanism [10]. For instance, Rodger et al. [31,32,33,34,35] had adopted MD to investigate the hydrate dynamic inhibitor, and discovered that the inhibitor was amphoteric, among which, the hydrophilic sulfur-oxygen group could suppress hydrate production, while the hydrophobic methyl could promote hydrate formation. Additionally, the supersaturated CH_4_ was to the benefit of hydrate synthesis, and the mechanisms of action of various hydrate catalysts were also different. The Natural Gas Hydrate Research Center of Guangzhou Energy Institute, Chinese Academy of Sciences [36,37,38], had discovered that, the decomposition of heating and hydrate inhibitor on hydrate began on the surface, which was gradually transmitted layer by layer from outside in, and the high hole occupancy and low temperature were beneficial for the stabilization of the methane hydrate crystal structure. Wu et al. [39] had discovered based on MD that, the yield limit of hydrate was greatly affected by temperature and grain size.

The actual hydrate system generally contains defects (such as cavity, polycrystal, and vacancy) and impurities (like sand sediment) [40], which will lead to certain differences in the performance and microscopic structure between the actual hydrate system and the complete hydrate system. The application of MD to investigate the mechanical property and microscopic structure evolution during the hydrate decomposition failure process can effectively avoid multiple problems in experimentally investigating the hydrate performance. Consequently, this paper had utilized the MD method to study the microscopic structure and mechanical property of the defect-containing sI hydrate.

## 2. Results and Discussion

### 2.1. Failure Process During Stretching

**Figure 1:**
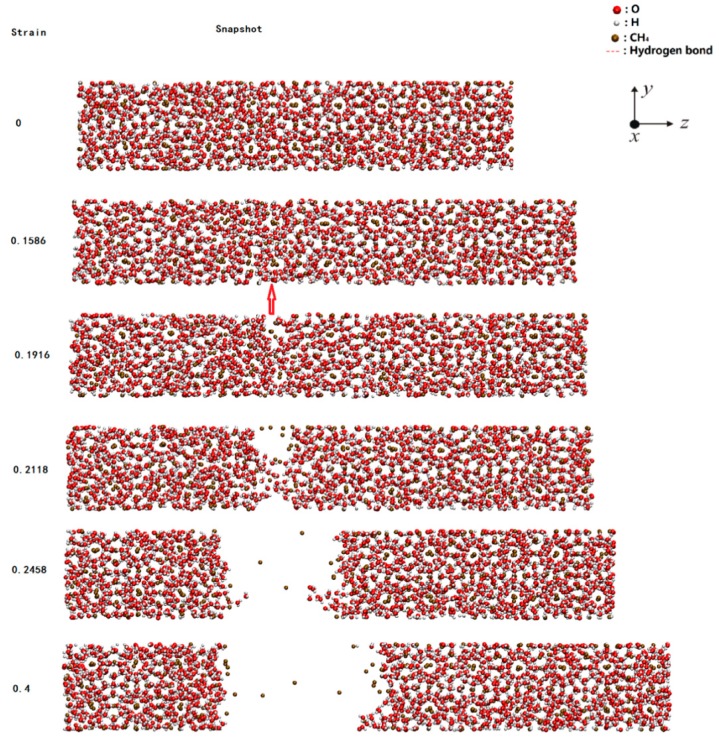
Snapshots of 2 × 2 × 10 (deletion percentage of 5.92%) system during stretching. (The arrow is the place where hydrate cracked).

Herein, Figure 1 shows the tensile strain process of the model with the percentage of randomly deleted particles of 5.92%, where X and Y directions represent 2 cell units, and Z direction indicates 10 cell units. At the initial stage of strain, the model still maintained the crystal structure of methane hydrate and it was subject to elastic strain, which could partially increase the model length at the tensile direction. With the increase in strain, crack appeared in the model, the crystal structure near the crack was destroyed, and the entire fault was uniform. By contrast, the crystal structure of methane hydrate remained unchanged in the area far away from the crack. After the appearance of a crack in the model, the strain at the tensile direction in the material gradually decreased. 

### 2.2. Strain–Stress Curves with Various Defect Ratios

**Figure 2:**
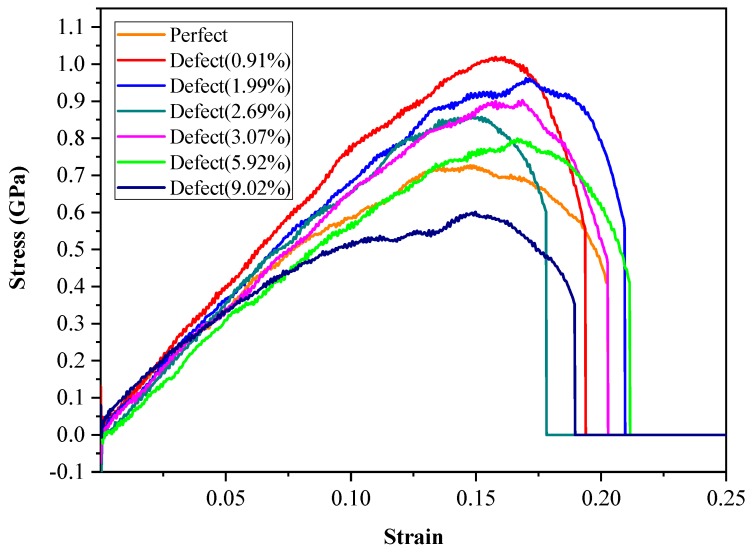
The strain–stress curves in the Z-axis of different deletion percentage of hydrate systems during stretching.

In Figure 2, the curve of the complete hydrate structure (denoted as “Perfect”) was consistent with the simulation results by Wu et al. [39]. Also, Young’s modulus of the studied curves agree with the experimental data [10]. The stress–strain curve of each system experienced three stages. The first stage was the linear elastic strain stage, in which the stress showed linearity with the strain. At this stage, the stress–strain curve of each system superposed each other, and it could be figured out based on a rough calculation of Young’s modulus in the experiment that the Young’s modulus of each model was similar. The second stage was the plastic deformation stage, in which the stress–strain curve of each system displayed non-linear changes; when the stress had reached the maximum value, the stress value of each model rapidly decreased within the limited variation range of strain, suggesting that each system had displayed the characteristics of brittle material failure. The third stage was the stage after failure, in which the stress value of each system remained zero with the increase in strain. 

It could be discovered when comparing the effects of various proportions of defects on the system mechanical property that there was no obvious rule regarding the effects of various defect proportions on the mechanical property with the reduction in total system particles. When the reduction of system particle number was <5.92%, the maximum system stress was reduced with the decrease in total particles, but it remained greater than the maximum stress of the 2 × 2 × 10 system, which indicated that the deletion of a certain proportion of particles in the system could partially enhance the mechanical property of hydrate. This might be because the microscopic structure energy of the particle-deleting part was optimized during the tensile process, and the motion of some water molecules had formed new hydrogen bonds, which could boost the mechanical property of the entire structure. When the system particle deletion proportion reached 9.02%, the stress-strain curve of the system was markedly reduced compared with that of the complete lattice, and its mechanical property was weakened. This was because excessive particles were deleted in the system, which had resulted in the incomplete structure of the methane hydrate system, along with the weak intermolecular forces on the tensile fracture surface. 

### 2.3. F3 Order Parameters

To intensively explore the relationship between the hydrate system microscopic structure and its mechanical properties, the order parameter was introduced to analyze the hydrate microscopic structure. The order parameters of the water molecule are generally used to depict the arrangement regularity of the water-containing system microscopically. F3 order parameter [41] is the parameter constituted by three water molecule structures, which represents the degree of tetrahedral arrangement formed by any one oxygen atom with other surrounding oxygen atoms, as expressed below (Equation (1)):

(1) $$F3=\left\{{\left(\cos\varphi_{jik}\left|\cos\varphi_{jik}\right|+\cos^{2}\left(104.52^{{}^{\circ}}\right)\right)}^{2}\right\}$$

where *ϕ_jik_* is the included angle formed by the specified oxygen atom *i* with the two oxygen atoms *j* and *k* in the adjacent region (assigned as the region that is 3.2 Å from the oxygen atom). The H–O–H included angle in the TIP4P water molecule is 104.52°. The F3 order parameter of normal water is about 0.1, while the hydrate can form the saturated hydrogen bond with the oxygen atom in the ice system as well as the water molecule where the adjacent oxygen atom is located, and its F3 order parameter is 0. The program for computing F3 order parameter was verified with Li et al. [42,43] works.

**Figure 3:**
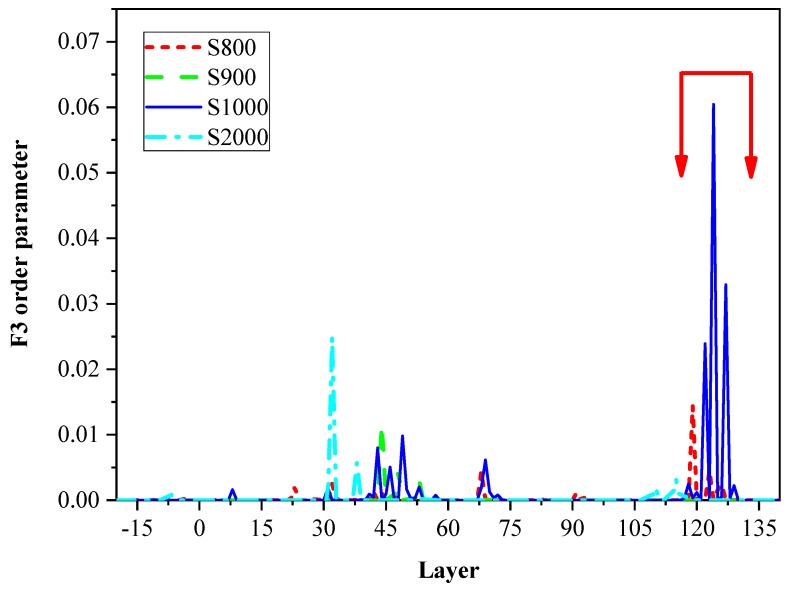
F3 order parameter for 2 × 2 × 10 unit cells of sI methane hydrate during stretching. (The arrows are the place where hydrate cracked).

The F3 order parameters of various defect systems were statistically analyzed, respectively, which focused on discussing the changes in F3 order parameter of the system before and after failure. Firstly, the 2 × 2 × 10 unit methane hydrate with complete lattice structure was analyzed, and the F3 order parameter output near the failure time step during the tensile process was shown in Figure 3.

The x-coordinate represented the size delamination of the system at the tensile direction, while the y-coordinate was the F3 order parameter. The changes in F3 order parameter of the system at the information output frames of 800, 900, 1000, and 2000 were analyzed (the failure occurred between 900–1000 frames). In the figure, the red arrow interval represented the failure location interval of the system microscopic structure in the Z-direction. As could be observed from Figure 3, at the output frames of 800 and 900, the F3 order parameter of the system remained almost unchanged, which fluctuated around 0, suggesting that the system had maintained the hydrate crystal structure. At the output frame of 1000, the F3 order parameter had fluctuated and peaked at 0.06, and it was close to that of normal water (0.1). Moreover, the Z-direction of the system with fluctuation had overlapped the fracture interval. At the output frame of 2000, the F3 order parameter of the system had little change in the Z-direction, which indicated that the part far away from the fracture maintained the hydrate crystal structure after failure. Upon comparison, the F3 order parameters of all the other structures (Supplementary Figure S1) had displayed a similar trend. 

## 3. Materials and Methods

### 3.1. Simulation Model and Computational Method

**Figure 4:**
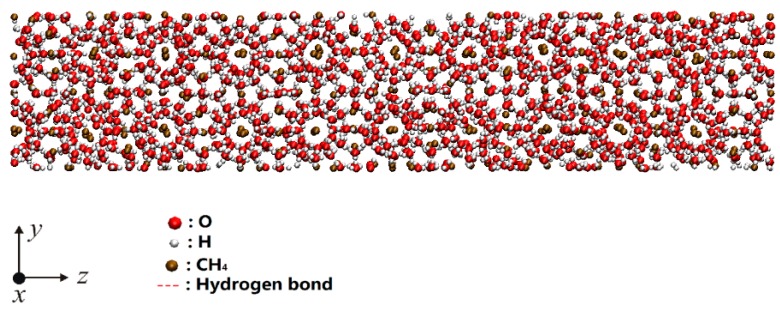
Simulation model for 2 × 2 × 10 unit cells of sI methane hydrate.

The sI hydrate was employed to carry out the mechanical tests and MD simulation in this study. sI methane hydrate is a type of the common hydrate, a unit cell of body-centered cubic (bcc) structure containing 46 water molecules. The hydrate crystal structure used in this paper was modified based on our previous study [42,43], and the defect-free sI hydrate stable structure of the 2 × 2 × 10 (X × Y × Z) unit cell system was constructed as the initial model, as shown in Figure 4. Subsequently, the particles were randomly deleted from the structure, to obtain the hydrate structures with various defect rates. Further details about the stable structure were described in [42,43]. In this paper, the sI hydrate of the complete 2 × 2 × 10 unit cell system was deleted to obtain the hydrate systems with various defects. 

In this study, the open-source LAMMPS software (15 Nov 2018 version, Sandia National Laboratories, Albuquerque, NM, USA) [44] is a large-scale atomic/molecular massively parallel simulator, which was adopted for simulation. The Lennard-Jones (LJ) form of hard sphere model [42,45] was utilized to describe the interaction between methane molecules, while the TIP4P model [46] was used to depict the physiochemical properties of water molecules. At the same time, the Lorentz–Berthelot compositing rules [47] were employed in this study to simulate the interaction among various particles in the system. 

### 3.2. Computational Parameters

**Figure 5:**
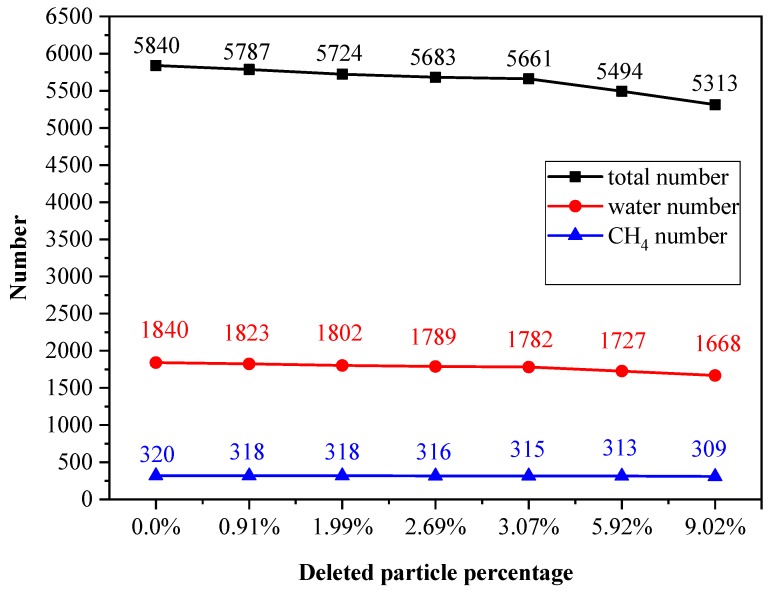
Deletion percentage of system particles for 2 × 2 × 10 unit cells of sI methane hydrate.

Parameters such as the total particle number, number of methane molecules, and number of water molecules are shown in Figure 5, where the x-coordinate represents the deletion percentage of system particles, and the y-coordinate indicates the total number of various particles.

The periodic boundary conditions were applied in the X, Y, and Z directions of the simulator, the Nose–Hoover algorithm was used to control the system temperature and pressure, the Velocity–Verlet algorithm [43] was utilized to solve the equation of motion of the particles, and the Ewald algorithm [48] was adopted to calculate the remote coulomb force of H_2_O, with the cutoff radius of 10 Å.

The systems after deletion of various percentages of particles were performed stable relaxation; firstly, the isothermal–isochoric (NVT) ensemble [49] (number of particles (N), volume (V), and temperature (T)) was relaxed for 1,000,000 steps at 200 K at the time step of 0.2 *fs*; subsequently, the isothermal–isobaric (NPT) ensemble [50] (number of particles (N), pressure (P), and temperature (T)) was relaxed under 200 K and 10 MPa conditions for 1,000,000, with the time step of 0.2 *fs*.

Afterward, the NPT ensemble was adopted for all test results at the constant temperature of 200 K; then, the test specimens were applied tensile strain along the Z-direction until failure at the time step of 0.2 *fs* and the strain rate during the tension of 1 × 10^7^/s. The system particle information was output at every 1000 steps, and the corresponding data were counted for analysis.

## 4. Conclusions

The study on molecular dynamics of methane hydrate involves the exploitation and application of methane hydrate technology, which attracted increasing worldwide attention. This study has utilized the MD method to investigate the influence of defect on the microscopic structure and mechanical property of the sI methane hydrate system based on the complete lattice hydrate. Additionally, the F3 order parameters are adopted to examine the microscopic structure of the sI methane hydrate before and after failure. Firstly, the 2 × 2 × 10 unit sI methane hydrate is selected as the initial model to construct the defect-containing hydrate structure. In addition, particle deletion at various ratios is performed on the initial model structure, and then the models with various defect proportions are subject to tensile strain, to analyze the evolution of microscopic structures and discover the mechanical property for each model system to maintain the brittle materials.

Subsequently, the stress–strain relationship of each system is calculated and analyzed, and it is discovered that the mechanical property of the system will be reduced at the system particle deletion rate of 9.02%. The maximum stress value of this system is lower than that of the initial model and will develop brittle failure prior to the initial structure; moreover, the mechanical properties of models at other defect ratios are partially enhanced compared with the initial model. Additionally, the microscopic structures of the sI methane hydrate before and after failure were analyzed by means of the F3 order parameter value method, the F3 order parameters near the crack will be subject to great fluctuations at the time of failure of the sI methane hydrate structure. Since the actual methane hydrate usually contains lattice defects or cavity and other impurities, the phenomenon and conclusions drawn in this study provide a basis for the study of the microscopic structure, mechanical characteristics, and practical exploitation of methane hydrate. Moreover, the structural properties and microscopic mechanism of the defect-containing hydrate will be further studied, and the experimental verification will be conducted in our future work.

## Supplementary Materials

Supplementary materials can be found at https://www.mdpi.com/1422-0067/20/9/2305/s1.
