# Supplementary material for: Molecular Simulation Study on the Microscopic Structure and Mechanical Property of Defect-Containing sI Methane Hydrate
Source: Int J Mol Sci. 2019 May 9;20(9):2305. doi: 10.3390/ijms20092305 (PMC6539317; doi:10.3390/ijms20092305)
Supplement: Supplementary file 1 [file ijms-20-02305-s001.pdf]

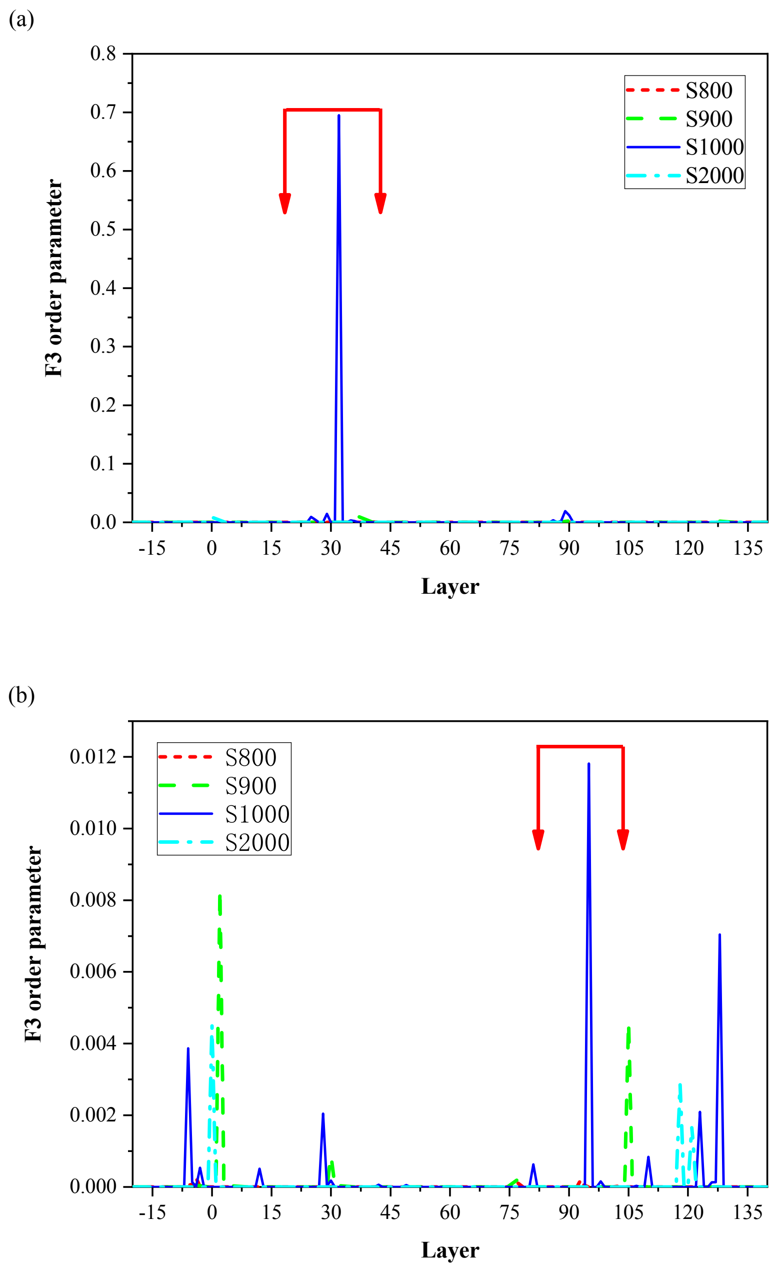
(a)
F3 order parameter
0.8
0.7
0.6
0.5
0.4
0.3
0.2
0.1
0.0
-15
0
15
30
45
60
75
90
105
120
135
Layer
S800
S900
S1000
S2000
(b)
F3 order parameter
0.012
0.010
0.008
0.006
0.004
0.002
0.000
-15
0
15
30
45
60
75
90
105
120
135
Layer
S800
S900
S1000
S2000

(c)

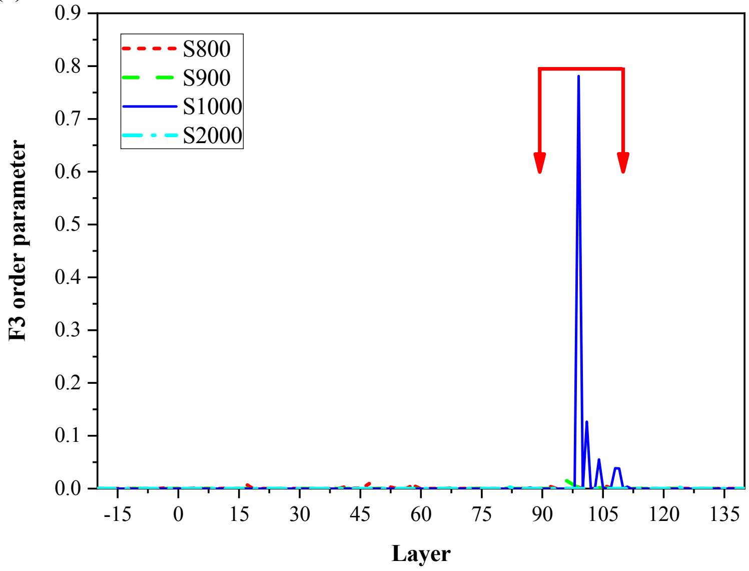

0.9
0.8
0.7
0.6
0.5
0.4
0.3
0.2
0.1
0.0
F3 order parameter
S800
S900
S1000
S2000
-15
0
15
30
45
60
75
90
105
120
135
Layer

(d)

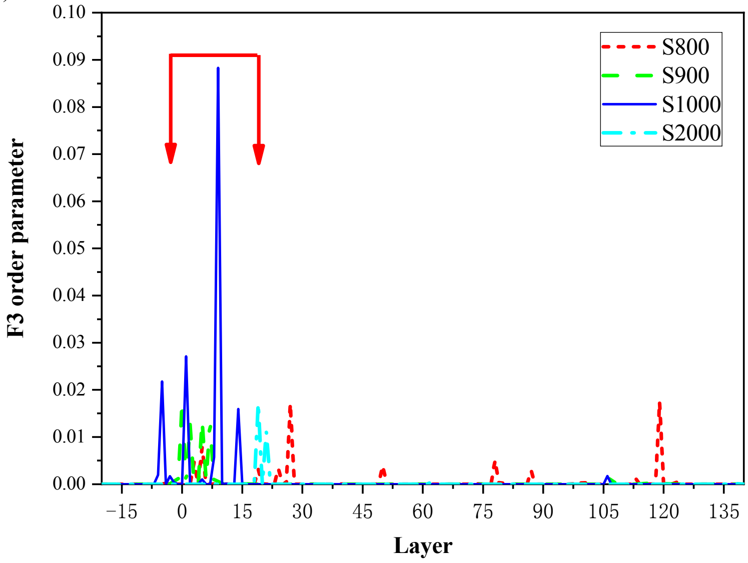

0.10
0.09
0.08
0.07
0.06
0.05
0.04
0.03
0.02
0.01
0.00
F3 order parameter
S800
S900
S1000
S2000
−15
0
15
30
45
60
75
90
105
120
135
Layer

(e)

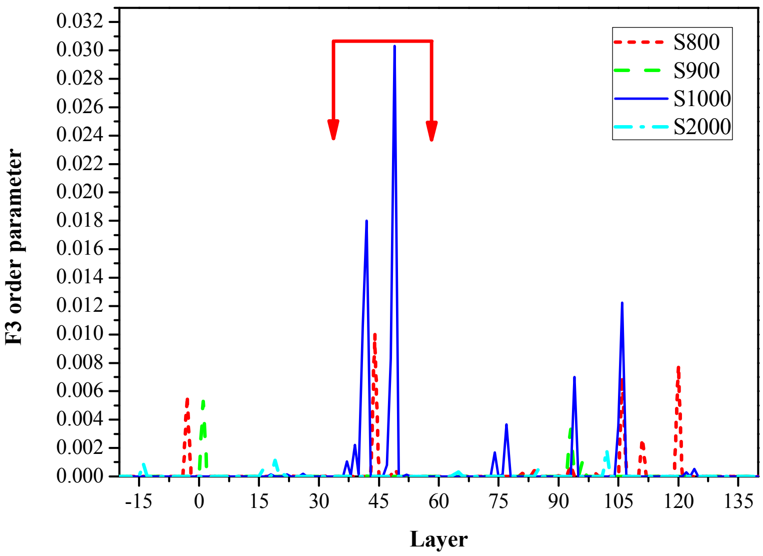

(f)

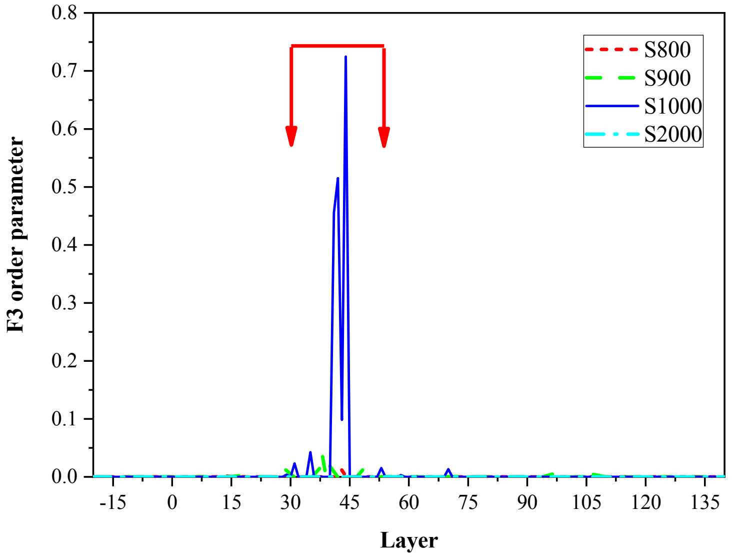

Fig. S1 F3 order parameter for 2×2×10 unit cells of different sI methane hydrate systems during stretching. (a) deletion percentage of 0.91%; (b) deletion percentage of 1.99%; (c) deletion percentage of 2.69%; (d) deletion percentage of 3.07%; (e) deletion percentage of 5.92%; (f) deletion percentage of 9.02%.
